# E6/E7 and E6^*^ From HPV16 and HPV18 Upregulate IL-6 Expression Independently of p53 in Keratinocytes

**DOI:** 10.3389/fimmu.2019.01676

**Published:** 2019-07-23

**Authors:** Cristina Artaza-Irigaray, Andrea Molina-Pineda, Adriana Aguilar-Lemarroy, Pablo Ortiz-Lazareno, Laura P. Limón-Toledo, Ana L. Pereira-Suárez, Wendoline Rojo-Contreras, Luis F. Jave-Suárez

**Affiliations:** ^1^Centro de Investigación Biomédica de Occidente, Instituto Mexicano del Seguro Social, Guadalajara, Mexico; ^2^Programa de Doctorado en Ciencias Biomédicas, Centro Universitario de Ciencias de la Salud, Universidad de Guadalajara, Guadalajara, Mexico; ^3^Centro Médico Nacional de Occidente, Instituto Mexicano del Seguro Social, Hospital de Ginecología y Obstetricia, Guadalajara, Mexico; ^4^Departamento de Fisiología, Centro Universitario de Ciencias de la Salud, Universidad de Guadalajara, Guadalajara, Mexico; ^5^Instituto Mexicano del Seguro Social, Hospital General Regional 110, Guadalajara, Mexico

**Keywords:** IL-6, E6^*^, cervical cancer, chronic inflammation, human papillomavirus, HPV16, HPV18

## Abstract

Keratinocyte infection with high-risk human papillomavirus genotypes has been linked to cancer development. In cervix, the alpha HPV16 and HPV18 have been reported as the mayor causative agents of cervical cancer. Oncogenic progression and chronic inflammation are closely related processes, with IL-6 as one of the main pro-inflammatory cytokines involved. However, there are limited studies about the regulation of IL-6 by low and high risk HPVs and the HPV proteins implicated in this modulation. In this work, we report the overexpression of IL-6 in HPV infected cervical cancer derived cell lines (HeLa and SiHa) compared to non-tumorigenic keratinocytes (HaCaT), and in Cervical Intraepithelial Neoplasia grade 1 HPV16 and HPV18 positive cervical samples compared to HPV negative samples without lesions. Moreover, we generated HaCaT keratinocytes that express E5, E6, and E7 from high risk (16 or 18) or low risk (62 and 84) HPVs. E5 proteins do not modify IL-6 expression, while E7 modestly increase it. Interestingly, E6 proteins in HaCaT cells upregulate IL-6 mRNA expression and protein secretion. Indeed, in HaCaT cells that express high risk HPV16E6 or HPV18E6 proteins, only the truncated E6^*^ isoforms were expressed, showing the stronger IL-6 overexpression, while in HaCaT cells that express low risk HPV62 and HPV84 full length E6 proteins, IL-6 was also upregulated but not so drastically. Since HaCaT cells have a mutated p53 form that is not degraded by the introduction of E6 or E6/E7, it seems that E6/E7 regulate IL-6 by an additional mechanism independent of p53. In addition, basal keratinocytes showed a strong expression of IL-6R using immunohistochemistry, suggesting an autocrine mechanism over proliferative cells. Altogether, IL-6 cytokine expression in keratinocytes is upregulated by E6 and E7 proteins from HPVs 16, 18, 62, and 84, especially by high risk HPV16 and HPV18 E6^*^, which may contribute to promote a pro-inflammatory and highly proliferative microenvironment that can persist over time and lead to cervical tumorigenesis.

## Introduction

Human Papillomaviruses (HPVs) are small non-encapsulated dsDNA viruses that show tropism for squamous epithelium therefore causing cutaneous or mucosal infections. Most HPV infections persist asymptomatically during all the lifetime; however, some of them can have clinical presentations from benign to malignant growth (1). Over 200 HPV genotypes have been identified and classified in taxonomic categories based on nucleotide sequence comparisons, genome organization, biology, and pathogenicity (2). *Alpha, Beta, Gamma, Mu*, and *Nu* genera hold all the HPVs described to date, and principally, the *Alpha* genus harbors all oncogenic HPVs associated to anogenital cancers (1). This subset of viruses has been extensively studied and they are referred to as high-risk HPVs (HR-HPVs) to distinguish them from the rest of HPV types, the low-risk HPVs (LR-HPVs), that commonly cause only benign epithelial lesions (3).

Cervical Cancer (CC) is the fourth leading cause of cancer deaths in women worldwide, and the second most frequent cancer in Mexico (4). HR-HPV persistent infection is the main etiological factor for CC development and HPV16 and HPV18 are the major HPV types associated to cervical carcinogenesis worldwide being therefore the best studied HPVs (5). Meantime, HPV62 and HPV84, considered as non-carcinogenic HPVs, are often found in cervical samples of Mexican women with different grades of lesions, but never in single infection in CC samples (6, 7). HR-HPV oncoproteins E5, E6, and E7 are the primary viral factors responsible for initiation and progression of CC. E6 and E7 proteins disrupt p53 and Rb functions, respectively, a crucial event for the cellular transformation, but additional important cellular targets have also been identified. Alterations on many cellular pathways lead to the overcoming of negative growth regulation by host cell proteins and to genomic instability which finally results in malignant progression (8). The comparison of HR-HPVs oncoproteins against their non-oncogenic LR-HPV counterparts has revealed that the limited activities of the LR-HPV proteins, like their inability to degrade Rb and p53, may have allowed these low risk viruses to send fewer distress signals and therefore to better coexist with their host cell (9, 10).

HPV gene transcription is controlled by two main promoters whose activation is regulated by the differentiation state of the infected cell. This results in the synthesis of polycistronic mRNAs, which are further regulated by alternative splicing processes (11). Alternative splicing within E6-E7 ORFs has been exclusively observed in HR-HPVs, not in LR-HPVs (12). When no splicing occurs within E6 ORF, full length E6 (fl-E6) protein is expressed, while E7 protein can be transcribed from mRNAs with or without splicing of E6. Moreover, for the splicing process in HPV16 E6-E7 mRNA, the spliceosome recognizes a donor splice site within E6 ORF and one of the different acceptor sites located in the early mRNA, leading to different shorter E6 mRNA transcripts called E6^*^, mainly E6^*^I (also called E6^*^) and E6^*^II, being E6^*^I the most abundant spliced E6 (13). Regarding HPV18, it appears to transcribe only one short E6 isoform, the E6^*^I (14).

On the other hand, an important player during the carcinogenic process is interleukin-6 (IL-6), a pleiotropic cytokine mainly reported as a pro-inflammatory molecule. IL-6 leads to inhibition of apoptosis in cells during inflammation through the activation of JAK-STAT signaling pathways after its binding to IL-6 receptor (IL-6R); however, as these pathways maintain cells progressing toward neoplastic growth, inflammation, and cancer are two closely related processes (15, 16). IL-6 is highly up-regulated in many cancers and is considered as a crucial cytokine during tumorigenesis. Therapeutic strategies targeting the IL-6 pathway are in development for cancers and inflammatory diseases. Regarding CC, high microenvironmental IL-6 levels promote angiogenesis and CC development (17). Also, IL-6 expression is higher in CC tissue compared to non-tumorigenic adjacent tissue and this overexpression is correlated with tumor size and poor prognosis (18). Furthermore, it has been reported that overexpression of IL-6 enhances the tumorigenic activity of basal cell carcinoma cells by inhibiting apoptosis and promoting angiogenesis (19). An interesting study on cytokine expression in HPV immortalized epithelial cells showed that in human keratinocytes immortalized with E6 and E7 genes from carcinogenic alpha HPV16 and beta HPV38, IL-6 mRNA expression and protein secretion was higher than in control cells (20), suggesting that the presence of E6/E7 from carcinogenic HPVs increases IL-6 expression. Interestingly, the target proteins of the high risk E6 and E7, p53, and Rb, regulate the expression of IL-6 negatively, raising the question whether degradation of those proteins are the main mechanisms of IL-6 induction by high risk HPVs. As we mention before low risk HPVs are unable to induce p53 and Rb degradation.

Therefore, the aim of this work was to evaluate the influence of HR HPVs 16 and 18 and LR HPVs 62 and 84 viral E6, E7, and E5 proteins on IL-6 expression in keratinocytes and whether this influence is mediated through p53.

## Materials and Methods

### Cell Culture and Cell Line

HaCaT, HeLa (HPV18+) and SiHa (HPV16+), Lenti-X 293T cells were cultivated in Dulbecco's Modified Eagle Medium (DMEM) with L-glutamine (584 mg/L), sodium pyruvate (110 mg/L), penicillin (100 U/ml), streptomycin (100 μg/ml), 10% Fetal Bovine Serum (FBS) and specific concentrations of D-glucose for each cell line (4.5 g/L for HaCaT and Lenti-X 293T cells, and 1 g/L for HeLa and SiHa cells) (Gibco, Thermo Fisher Scientific, Waltham, MA). Lenti-X 293T cells were grown in medium supplemented with Tet-Free FBS (Clontech, Mountain View, CA). hTERT-scrambled and hTERT-crisp53 (primary human keratinocytes immortalized with hTERT and then silenced for p53 with Crispr/Cas9 system) were cultivated as previously described and Feeder layer was prepared by treating NIH 3T3 cells with Mytomycin C (0.5 mg/ml) for 2 h (21).

### Cloning of E5, E6, and E7 From HPV16, 18, 62, or 84

E5, E6, and E7 Open Reading Frames (ORFs) from HPV16, 18, 62 or 84 were amplified by PCR from genomic DNA (gDNA) extracted from cervical biopsies of women infected with those viruses with primers described in Table S1. PCR experiments were done using *Expand High Fidelity PCR System kit* (Sigma-Aldrich, Toluca, Mexico), PCR products were separated by agarose gel electrophoresis and the bands of interest were purified with *Wizard SV Gel and PCR Clean-Up System kit* (Promega, Madison, WI). Each PCR product was first cloned in the pGEM-T Easy vector (Promega). For HPV18E5, a synthetic DNA fragment flanked with EcoRI restriction sites (gBlock) was constructed using the sequence of HPV18E5 as template (*Integrated DNA Technologies*, Coralville, IA) and cloned into the pGEM-T Easy vector. Then, the ORFs of E5, E6, and E7 were subcloned in the expression vector pLVX-Puro (Clontech) using EcoRI enzyme for restriction, Antarctic Alkaline Phosphatase for vector dephosphorylation and T4 DNA ligase for ligation (New England Biolabs, Ipswich, MA), following the provider instructions. Cloned genes were sequenced using *BigDye® Terminator Cycle Sequencing kit* (Applied Biosystems, Thermo Fisher Scientific) and *ABI PRISM 310 Genetic Analyzer*. The obtained sequences were aligned with reference sequences reported in the NCBI by using the *CLC MainWorkbench7.0* software (Qiagen Bioinformatics, Redwood City, CA). Plasmids obtained on this way were pLVX-16E5, pLVX-16E6, pLVX-16E7, pLVX-18E5, pLVX-18E6, pLVX-18E7, pLVX-62E5, pLVX-62E6, pLVX-62E7, pLVX-84E5, pLVX-84E6, and pLVX-84E7. These plasmids were further used to produce lentiviral particles.

### Production of Lentiviral Particles Carrying Viral Genes and Infection of HaCaT Cells

Lenti-X 293T cells were transfected with each of the 12 pLVX expression vectors and the pLVX-Puro empty vector using the *Lenti-X HTX Packaging System* (Clontech) and Lipofectamine 2000 transfection reagent (Thermo Fisher Scientific). Cell supernatants were collected following the *Lenti-X Lentiviral Expression System* (Clontech) instructions and the presence of lentivirus was confirmed with *Lenti-X GoStix*™ (Clontech). HaCaT cells were individually infected with the lentiviral particles containing each viral gene and selected with 0.5 μg/ml of Puromycin antibiotic for approximately 2 weeks until control cells (HaCaT cells without any infection) were death. The transduced HaCaT cells HaCaT-pLVX, HaCaT-16E5, HaCaT-16E6, HaCaT-16E7, HaCaT-18E5, HaCaT-18E6, HaCaT-18E7, HaCaT-62E5, HaCaT-62E6, HaCaT-62E7, HaCaT-84E5, HaCaT-84E6, and HaCaT-84E7 were grown in the same conditions as the parental HaCaT cell line.

### Quantitative PCR Analysis

RNA was extracted with *RNeasy Plus Mini Kit* (Qiagen, Cat. No. 74136) or *All Prep DNA/RNA Mini kit* (Qiagen, Germantown, MD) and cDNA was obtained with *Transcriptor First Strand cDNA Synthesis Kit* (Roche, Pleasanton, CA) or *Revert Aid H Minus First Strand cDNA Synthesis Kit* (Thermo Fisher Scientific) following the manufacturers' instructions. Quantitative PCR (qPCR) was performed using the *LightCycler 2.0* platform (Roche) and the *LightCycler FastStart DNA Master plus SYBR Green I Kit* (Roche). In addition, the *Agilent Technologies Stratagene Mx3005P* equipment (Serial No. DE10901331) and the *MESA GREEN qPCR MasterMix Plus for SYBR Assay* (Eurogentec, Liege, Belgium) were used. β-actin, GAPDH, RPLP0 and RPL32 were used as reference genes. The results were analyzed with *Light Cycler Software 4.1* or the *MxPro qPCR Software*. E5, E6, and E7 qPCR products were visualized in 2% agarose gels. Primers used to amplify HPV16 E6/E6^*^ were the following: Fwd ACTGCAATGTTTCAGGACCCA and Rev TCAGGACACAGTGGCTTTT, the product size was 343 bp for fl-E6 and 161 bp for E6^*^. All additional primer pairs used in this work are described in Table S1.

### Western Blot and ELISA Test

For protein detection by western blot, 50 μg of protein were separated in a polyacrylamide *Mini-PROTEAN TGX Gel* (Bio-Rad, Hercules, CA) and transferred to a PVDF membrane by semi-dry transfer using the *iBlot Dry Blotting System* (Thermo Fisher Scientific). The membrane was blocked for 1 h at 37°C with *PBS Odyssey Blocking Buffer* (Li-COR Biosciences, Lincoln, NE). Then, primary antibody was added and the membrane was incubated overnight at 4°C: Anti-p53 mouse monoclonal IgG2a (Santa Cruz Sc-126, 1:200 dilution) or Anti-actin (I-19) Goat polyclonal IgG (Santa Cruz Sc-1616, 1:4,000 dilution). After washing with *1x PBS (*phosphate-buffered saline) *Tween*, the membrane was incubated for 1 h at 37°C with a fluorophore labeled secondary antibody: Anti-mouse donkey polyclonal IgG 800CW (926-32212, Li-COR, 1:15,000 dilution) or Anti-goat donkey polyclonal IgG 680RD (926-68074, Li-COR, 1:15,000 dilution). Finally, after washing, the membrane was scanned with the *Odyssey Imaging System* (Li-COR). ELISA experiments were performed following the manufacturer's instructions. IL-6 protein was measured in HaCaT, HaCaT-pLVX, HaCaT-16E6, HaCaT-18E6, HaCaT-62E6, HaCaT-84E6, HeLa, and SiHa cells supernatants using the *Human IL-6 ELISA Kit* (Thermo Fisher Scientific).

### Cell Transfections

Transfections were conducted in HaCaT cells using the *X-tremeGENE 9 transfection reagent* (Roche), 3 μL of transfection reagent was used for each ug of DNA, all other transfection procedures were as recommended by the manufacturer. Twenty-four hour post-transfection, cells were harvested and RNA was extracted. HaCaT-pLXSN and HaCaT-16E6E7 stable transfectant cells were established as follow: 3 × 10^5^ HaCaT cells were seeded in 6 well plates, and 24 h later, the cells were transfected with 1 μg of pLXSN vector or pLXSN-16E6E7 vector. Twenty-four hour post-transfection, 500 μg/mL of G-418 antibiotic was added to the cells. The cells were maintained growing with antibiotic for 23 days and then RNA was extracted to perform the qPCR assays.

### Cervical Sample Collection and Analysis

Cervical samples were collected from women who attended a medical examination at the Clinic of Dysplasia in the Gynecology and Obstetrics Hospital- Centro Médico Nacional de Occidente (CMNO-IMSS) in Guadalajara, Jalisco, Mexico. Before sampling, all participants were informed about this research protocol and decided to participate voluntarily; all of them signed informed consent. All procedures performed were in accordance with the ethical standards and were approved by the National Scientific Research Committee of IMSS (CNIC) with the Register number: R-2012-785-090. Sample recruitment was done from 2017 to 2018 by gynecologists, with a cytobrush inserted into the endocervical canal and positivity to HPV16 and 18 was determined by the Linear Array Genotyping Test as previously described (7). We recruited 9 HPV negative cervical samples from women without lesion (control), 7 HPV16 positive cervical samples from women with Cervical Intraepithelial Neoplasia grade 1 (CIN1) lesions, and 3 HPV18 positive CIN1 samples, and RNA was extracted from those samples to perform qPCR analyses.

### Immunohistochemistry Analysis

Histologically normal cervical epithelium (*n* = 3) and squamous cell carcinoma samples positive to HPV16 (*n* = 3) that were paraffin-embedded were used for automated immunodetection assays using the Ventana BenchMark XT System, (Roche, Mannheim, Germany), the anti-IL-6R antibody (Santa Cruz Biotechnology, sc-373708, 1:100 dilution), and the ultraView Universal DAB Detection Kit (Roche Applied Science), following the manufacturers' instructions. IL-6R expression was evaluated and classified according to their level of positivity.

### Statistical Analysis

Statistical significance was determined using the unpaired t Student test assuming same standard deviation and Gaussian distribution. The results that were statistically significant are indicated with ^*^ when *p* < 0.05 and with ^**^ when *p* < 0.01 in the corresponding figures.

## Results

### IL-6 Is Overexpressed in HPV16 and HPV18 Positive CC Cell Lines and CIN1 Samples

**Figure 1:**
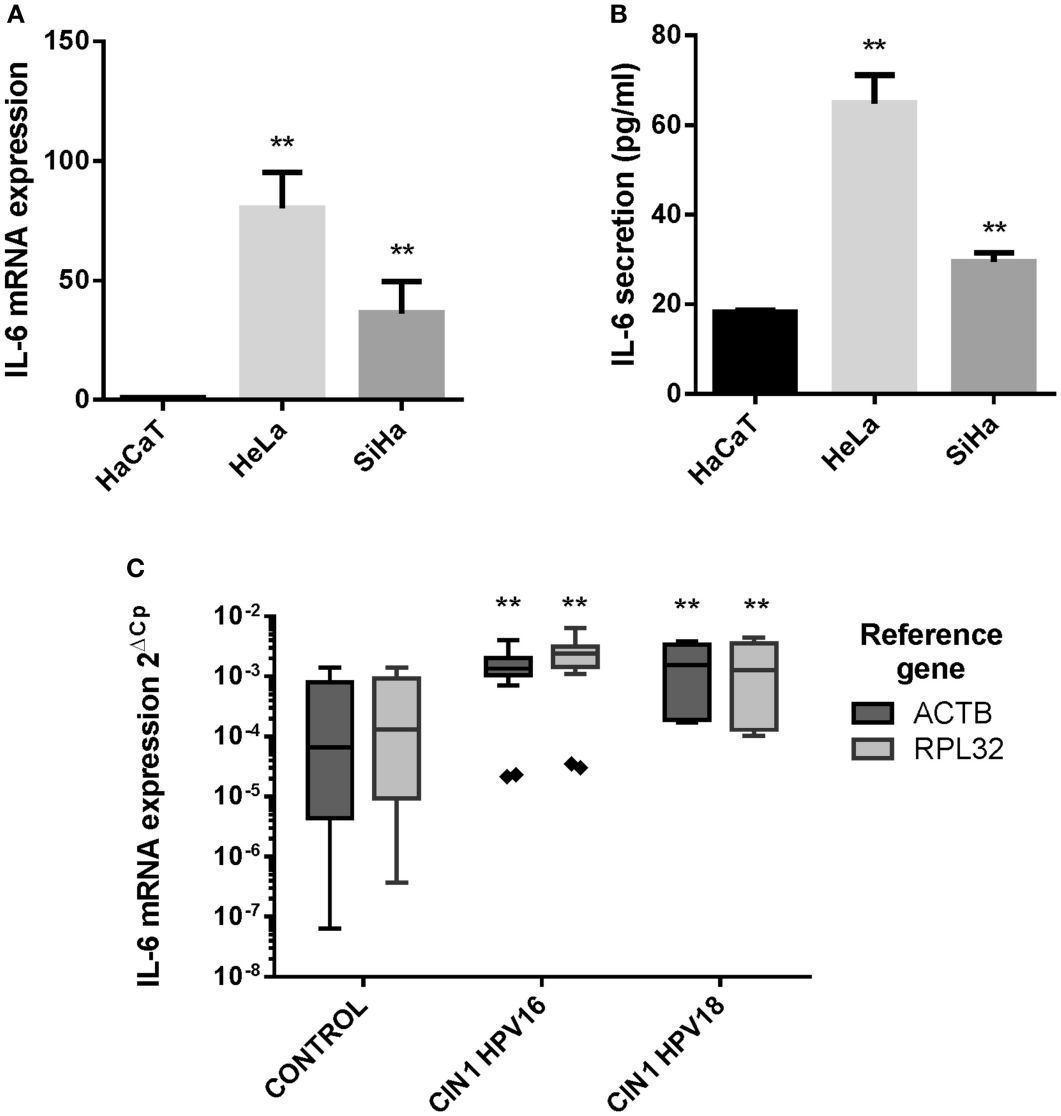
IL-6 expression in HaCaT, HeLa, SiHa cells, and in cervical samples. **(A)** IL-6 mRNA relative expression in HaCaT, HeLa, and SiHa cell lines evaluated by qPCR using actin, RPLP0 and RPL32 as reference genes. **(B)** IL-6 protein expression measured by ELISA test in HaCaT, HeLa and SiHa supernatants. Error bars in **(A,B)** represent standard deviation. **(C)** IL-6 mRNA expression in 9 HPV negative cervical samples without lesion (Control), 7 HPV16 positive CIN1 cervical samples (CIN I HPV16) and 3 HPV18 positive CIN1 cervical samples (CIN I HPV18) using actin and RPL32 as reference genes. Values are represented as 2^ΔCp^ (Cp Reference Gene—Cp Target Gene). The graph displays median (white lines), 25–75th percentile (boxes), interquartile ranges (Whiskers) and outlier data (triangles). Asterisks indicate statistical significance (**p < 0.01).

Initially, IL-6 expression was evaluated in HaCaT keratinocytes (HPV–) and HeLa (HPV18+) and SiHa (HPV16+) CC derived cell lines showing that IL-6 mRNA and protein is highly expressed in HeLa and SiHa cells compared to HaCaT cells (Figures 1A,B). As this result suggests that HR-HPVs may be promoting the overexpression of IL-6, IL-6 mRNA expression was evaluated in CIN1 HPV16 and HPV18 positive samples compared to control HPV negative cervical samples without lesion. The experiment reveals that in CIN1 samples positive for HPV16 or HPV18, IL-6 is upregulated compared to control samples (Figure 1C).

### IL-6 Expression Increases in Keratinocytes by the Presence of HR-HPV E6 and E7 Oncogenes, Especially E6^*^

**Figure 2:**
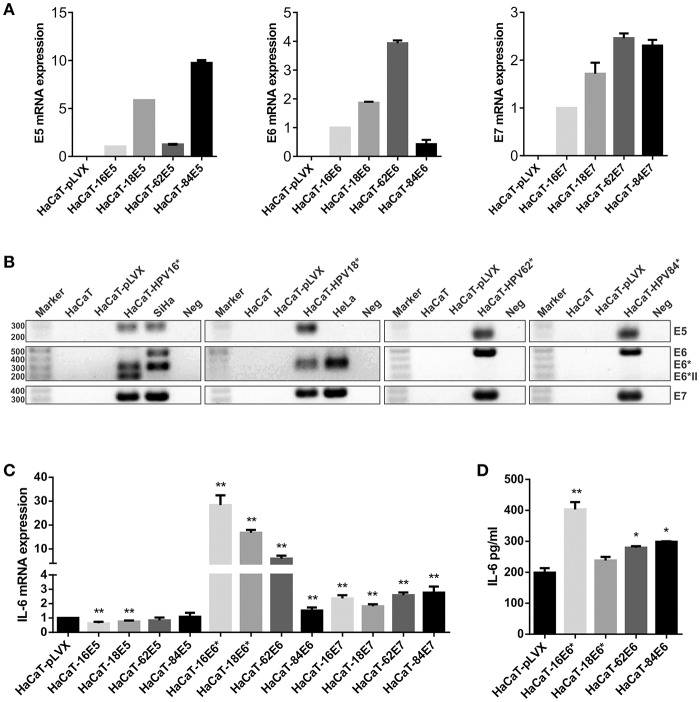
Evaluation of IL-6 expression in E5-, E6-, and E7-transduced HaCaT keratinocytes. **(A)** E5, E6, and E7 expression measured by qPCR in HaCaT transduced cell lines using HaCaT-16E5, HaCaT-16E6, and HaCaT-16E7 as calibrators and actin as reference gene. **(B)** Visualization of E5, E6, E7, and E6 isoforms mRNAs expressed in transduced HaCaT cell lines, HeLa, and SiHa cells. (*) HaCaT-16, HaCaT-18, HaCaT-62, and HaCaT-84 refer to HaCaT cells transduced with one of the 3 genes E5, E6, or E7 separately to check E5, E6, and E7 expression, respectively. The expected amplicon size for each viral gene is indicated in Table S1. Water was used as template in the negative controls (Neg). **(C)** IL-6 mRNA expression evaluated by qPCR in E5, E6, or E7 from HPV16, 18, 62, or 84 transduced HaCaT cells, using HaCaT-pLVX cells as calibrator and actin, RPLP0, and RPL32 as reference genes. **(D)** Protein levels of IL-6 measured by ELISA test in HaCaT-pLVX and HaCaT-E6 supernatants. Asterisks indicate statistical significance (*p < 0.05 and **p < 0.01).

The previous results indicate a possible role of HPV infection on the expression of IL-6, but whether this upregulation is a direct action of the oncoproteins or if oncogenic and non-oncogenic HPVs have the same ability to modulate IL-6, were new questions that needed to be addressed. In order to determine which HPV oncoprotein was involved in the regulation of IL-6 expression, the effect of E5, E6, and E7 oncoproteins from HPV16 and HPV18 compared to non-carcinogenic HPV62 and 84 proteins was evaluated. We decided to use the HaCaT cell line as study model since those cells have an inactive p53 protein and we wanted to evaluate the effect of the oncoproteins on IL-6 independently of its action over p53. It has already been reported that p53 negatively regulates IL-6 expression and that E6 induces the degradation of p53. First, we transduced HaCaT cells with E5, E6, or E7 from HPV16, 18, 62, or 84 individually. To evaluate transduction efficiency, the expression of the viral genes in the transduced cells was assessed with qPCR assay confirming that E5, E6, and E7 genes were expressed in the corresponding cell line compared to the HaCaT-pLVX control cells that do not express any of those genes (Figure 2A). Then, to corroborate those results, the qPCR amplification products were visualized in agarose electrophoresis. As observed in Figure 2B, E5 gene is expressed in HaCaT-16E5, HaCaT-18E5, HaCaT-62E5, HaCaT-84E5, and in SiHa (HPV16E5) cells, but not in HeLa cell line. E6 gene expression products reveal that HaCaT-16E6 expresses only the spliced isoforms of E6, E6^*^I, and E6^*^II, while SiHa cells express fl-E6 and E6^*^I. Moreover, both HaCaT-18E6 and HeLa cells express E6^*^ but no HPV18 fl-E6. Fl-E6 is expressed in HaCaT-62E6 and HaCaT-84E6 cells as no spliced isoforms of E6 have been reported in non-carcinogenic HPVs. Finally, E7 gene is expressed in the four HaCaT cell lines transduced with HPV16E7, HPV18E7, HPV62E7, or HPV84E7, as well as in HPV16 and HPV18 positive CC cells SiHa and HeLa, respectively.

After this characterization of our study model, IL-6 expression was evaluated in all transduced HaCaT cells compared to HaCaT-pLVX control, revealing an IL-6 mRNA overexpression in HaCaT-E6 cells, especially in HaCaT-16E6^*^ and HaCaT-18E6^*^ (Figure 2C). Also, IL-6 expression was around twice in HaCaT-E7 cells compared to HaCaT-pLVX, whereas there was a slight decrease in IL-6 mRNA levels in the presence of HPV16 and 18 E5, and no significant changes with HPV62 and 84 E5 (Figure 2C). As the most pronounced overexpression of IL-6 is observed in HaCaT cells that express the different E6 genes, we measured IL-6 protein in HaCaT-E6 supernatants with ELISA test. A higher secretion of IL-6 in HaCaT-E6 cells, especially in HaCaT-16E6^*^ cells was detected (Figure 2D).

**Figure 3:**
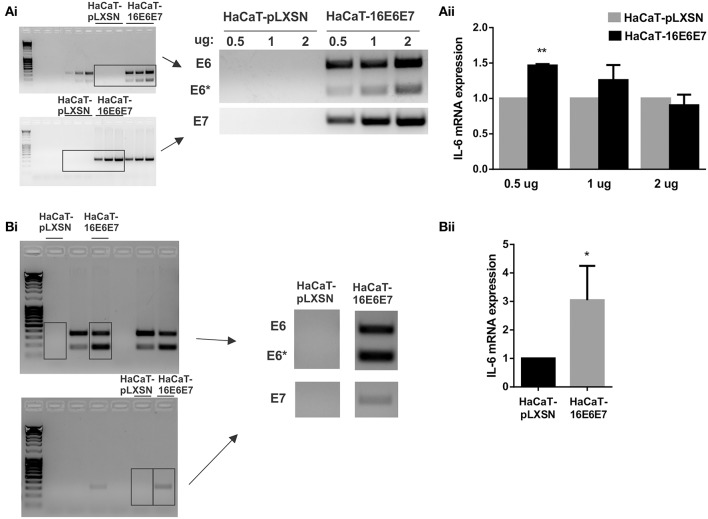
Evaluation of IL-6 expression in the presence of HPV16E6E7. **(Ai)** Expression of E6/E6* and E7 in transiently transfected HaCaT cells with 0.5, 1, or 2 ug of pLXSN or pLXSN-16E6E7 plasmids. Originals blots are shown left and representative pictures right. **(Aii)** IL-6 mRNA expression in those same cells. **(Bi)** E6/E6* and E7 expression in stable transfected HaCaT cells. Original blots left and representative pictures right. **(Bii)** IL-6 expression measured by qPCR in HaCaT and HaCaT transduced with E6/E7 from HPV16 (*p < 0.05 and **p < 0.01).

As HPV16E6^*^ strongly upregulates IL-6 expression, we focused on HPV16 oncoproteins and we did dose dependent transfections of HaCaT cells with pLXSN-HPV16E6E7 plasmid that expresses both HPV16 E6 and E7 oncoproteins. The transfected keratinocytes with 0.5, 1 or 2 ug of vector express E6 and E7 proteins (mainly fl-E6 but also E6^*^) and their expression levels are proportional to the plasmid dose (Figure 3Ai). IL-6 mRNA expression levels are also proportional to the vector dose but IL-6 overexpression in E6E7 transfected cells is not observed with 1 and 2 ug transfection, and is significantly higher with 0.5 ug transfection but not as drastic as in transduced cells (Figure 3Aii). Therefore, we established a stable transfectant HaCaT cell line that expresses both HPV16 E6 and E7 genes to check if IL-6 overexpression is time dependent and needs a longer exposition to the oncoproteins. E6, E6^*^I, and E7 oncogene expression is confirmed in HaCaT-16E6E7 stable transfectant cells (Figure 3Bi) and IL-6 expression increase in keratinocytes with E6 and E7 is corroborated with this model (Figure 3Bii).

### IL-6R Is Expressed in the Proliferative Basal Layer of Normal Keratinocytes and in Cervical Tumoral Cells

**Figure 4:**
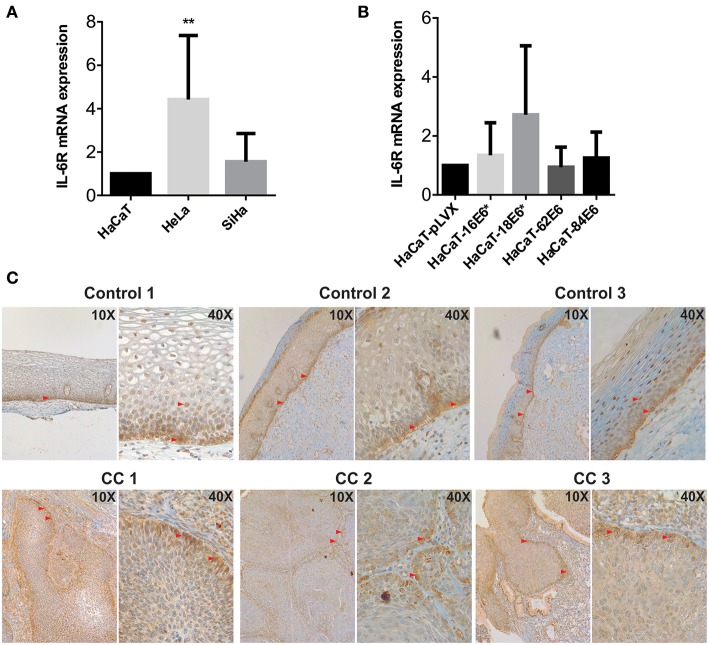
Evaluation of IL-6R expression in cell lines and cervical tissues. **(A)** IL-6R mRNA expression evaluated by qPCR in HaCaT, HeLa, and SiHa cells and **(B)** in E6 transduced HaCaT cells using HaCaT-pLVX as calibrator. Actin and RPL32 were used as reference genes, error bars represent standard deviation and asterisks indicate statistical significance (**p < 0.01). **(C)** IL-6R protein expression by immunohistochemical analysis in control and cervical cancer (CC) tissues. Red arrows indicate positive signal to IL-6R.

Considering the previous observations, we wondered if the keratinocytes also have a strong IL-6 receptor (IL-6R) expression so that IL-6 signaling could be autocrine. At mRNA level, HeLa and SiHa CC cells showed higher IL-6 expression compared to non-tumorigenic HaCaT cells (Figure 4A). In addition, we observed a modest tendency to increase in the expression of IL6R in HaCaT cells transduced with HPV16-E6^*^ and HPV18-E6^*^ compared to HaCaT-pLVX control cells (Figure 4B). Immunohistochemical analysis of cervical samples derived from women without lesion (control) and from women with CC (all CC samples were squamous cell carcinomas positive to HPV16 as confirmed by the Linear Array Genotyping test) showed that control tissues expressed IL-6R mainly in the proliferative basal layer of the epithelium. However, as the cells migrate to the parabasal and intermediate layers, a clear nuclear staining was observed (Figure 4C, control). Moreover, all CC tissues were IL-6R positive, especially in the tumor front (Figure 4C, CC). These results suggest that IL-6R expression is related to a proliferative phenotype in keratinocytes and is therefore expressed by cervical tumoral cells. The later could indicate a higher receptivity to IL-6 cytokine and its association with cell proliferation.

### Regulation of IL-6 by E6/E7 Is in Part p53 Independent

**Figure 5:**
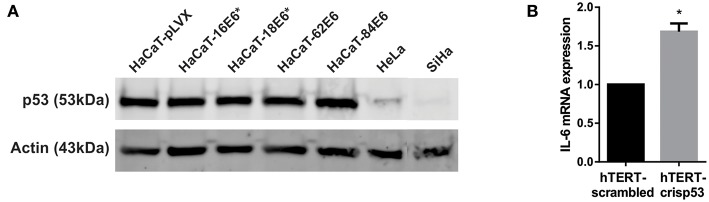
Effect of p53 on IL-6 expression. **(A)** p53 protein levels evaluated by western blot in HaCaT-pLVX, HaCaT-E6, HeLa, and SiHa cells. Boxes in the upper blot include wells relevant for this experiment, lower edited picture shows the same but in a clearer form. **(B)** IL-6 expression in hTERT immortalized keratinocytes silenced for p53 (hTERT-crisp53) compared to the control hTERT-scrambled cells. IL-6 expression was measured by qPCR. Actin was used as reference gene. The asterisk indicates statistical significance (*p* < 0.05).

HaCaT immortalized keratinocytes have mutations in p53 that affect its function and E6-targeted degradation (22, 23). It has previously been described that wild type p53 represses IL-6 promoter activity and that mutated p53 increase them (24–26). In order to evaluate p53 participation in E6 regulation of IL-6 expression, we first evaluated p53 protein degradation in HaCaT cells transduced with E6 of HPV-16,−18,−62, and−84. All four HaCaT-E6 transduced cell lines and HaCaT-pLVX control cells express p53 compared to HeLa and SiHa cells, in which p53 protein is degraded (Figure 5A). Moreover, despite of mutant p53 presence in HaCaT cells, E6E7 proteins increase almost three times IL-6 expression as already mentioned (Figure 3Bii), indicating an additional mechanism independent of p53 function. To corroborate IL-6 regulation by p53, IL-6 expression was evaluated in hTERT immortalized primary keratinocytes that have wt p53 or silenced p53 (hTERT-crisp53). As depicted in Figure 5B, there is an increase in IL-6 expression in the absence of p53. Additionally, it is worth mentioning that a previous study assessed IL-6 expression in primary human foreskin keratinocytes (therefore with p53wt) infected with the empty retroviral vector pLXSN (negative control) or with pLXSN vector expressing E6E7 genes from HPV16, and the results indicate a more pronounced induction of IL-6 expression in the presence of HPV16 oncoproteins (20).

## Discussion

Cytokines are soluble mediators known to keep immune homeostasis and participate in inflammatory response to infections or injury, they play diverse functions and can show pleiotropic properties. In particular, interleukin 6 participates in many biological processes such as chronic inflammation, autoimmunity, infectious diseases and cancer (27). The link between IL-6 and oncogenic progression has been widely reported and IL-6 overexpression has been observed in a variety of solid tumors, both in the tumor microenvironment and in the serum of affected patients, which explains why this cytokine has been proposed as a prognostic biomarker and a therapeutic target in cancer (28, 29). At early stages of HPV infection, a down-modulation of IL-6 expression and other cytokines, like IL-1β and IL-1α, has been reported (30). However, high expression of IL-6 has been related with a bad prognosis for cervical cancer patients and immunohistochemistry experiments demonstrate that tumor cells and stromal cells show high and moderate IL-6 expression respectively, and macrophages showed positive correlation with IL-6 positivity in stroma (31). In fact, different works have reported an upregulation of IL-6 in CC, but whether HPV oncoproteins are involved in this upregulation has been poorly studied. In addition, differences between HR-HPVs and LR-HPVs regarding the modulation or regulation of IL-6 expression has not previously been addressed.

In this work, we report the overexpression of IL-6 in HeLa and SiHa HPV positive CC cell lines compared to non-tumorigenic HaCaT keratinocytes. This result may suggest that CC derived cells have acquired the ability to up-modulate IL-6 expression. Audirac-Chalifour et al. working with samples at various stages of cervical cancer, observed the upregulation of IL-6 in low-grade squamous intraepithelial lesion and CC samples (32). In line with these observations, we report an IL-6 upregulation in HPV16 and HPV18 positive cervical samples from women with CIN1 compared to control cervical samples without lesion and negative to HPV infection. These results strongly suggested that HPV infection could mediate IL-6 regulation. Cheng et al. have observed an up-regulatory effect of HPV16 and HPV18 E6 and E7 to IL-6 expression in E6 and E7 transfected lung cancer cells (33). Also, the repression of HPV18 E6 and E7 in basal cell carcinoma cells reduced IL-6 expression levels (34). In addition, Dell'Oste et al. reported an increase in IL-6 mRNA expression and protein secretion in primary keratinocytes immortalized with both E6E7 genes from HPV16 compared to control keratinocytes (20). In this work we compare the effect of HR vs. LR viral proteins on IL-6 expression in HaCaT transduced cells, revealing an IL-6 upregulation by HR HPV16 and HPV18 E6^*^ and E7 oncoproteins individually, mainly by E6^*^. LR HPV62 and HPV84 E6 and E7 proteins, that have been barely studied, also induce the increase of IL-6 mRNA expression, even though the effect was not so pronounced as with HR oncoproteins. Interestingly, HPV16 and HPV18 E5 proteins decrease IL-6 expression whereas no significant variations were seen with HPV62 and HPV84 E5. IL-6 protein secretion was similar in HaCaT-18E6^*^, −62E6, and −84E6, and the higher IL-6 levels were observed in HaCaT-16E6^*^.

The analysis of viral gene expression in each transduced HaCaT cell line with E5, E6 or E7 from HPV16, 18, 62 or 84 demonstrated that exclusively E6^*^ spliced isoforms are expressed in HaCaT-16E6 and HaCaT-18E6 cells. Also, SiHa cells (HPV16+) expressed E5, E6, E6^*^, and E7 proteins, while HeLa cells (HPV18+) expressed only E6^*^ and E7 proteins and no E5 or fl-E6 expression was observed.

Transiently transfected HaCaT cells with HPV16E6E7 expressed both E6 and low levels of E6^*^ proteins and IL-6 expression was not significantly altered, however, in HaCaT-16E6E7 stable transfectant cells, both E6 and E6^*^ are expressed, predominantly E6^*^, and IL-6 is now overexpressed. The later suggests that the overexpression of IL-6 is time and E6^*^ expression dependent, and E6^*^ must prevail over fl-E6 to induce IL-6 upregulation. Following the last hypothesis, E6^*^ expression has been reported to be modulated by EGF and EGFR (35), and it is well established that the EGFR is mainly expressed in the basal layer of cervical epithelium (36). At early stages of HPV infection, when the viral genome remains episomal, the E6 mRNA is expressed at low levels and since the microenvironment of the basal layer is rich in EGF and EGFR, the expression of fl-E6 could be favored leading to the negative regulation of IL-6. Thereafter, the expression of E6^*^ could be induced by cell migration into the upper layers where less EGFR expression has been observed. Another possibility is that the loss of E2 regulation and the increment of E6 mRNA levels (37) could also favor E6^*^ expression. Whatever the mechanism involved, the final consequence is the increment of IL-6 expression and the induction of its connate pathway, which has been involved with tumor promotion (28, 38). IL-6 induction suggests E6^*^ as a pro-inflammatory oncogene, while other works have pointed out the alternate E6^*^I protein as the most abundant oncogene transcript in premalignant or malignant cervical and oropharyngeal tumors (39–41). In addition, the isoform E6^*^II has been also reported in CC and the association between E6^*^I/E6^*^II expression and the grade of cervical lesions are controversial (13). However, there are also studies that propose E6^*^II expression as an indicator of cervical neoplasia severity (42). Whether HPV16 E6^*^II by its own also regulates IL-6 expression remains unclear. Further studies need to be performed to better understand the E6 splicing patterns according to lesion severity. Indeed, many works have tried to elucidate the role of those fl-E6 and E6^*^ spliced proteins in carcinogenesis, but there is not a general consensus on their biological significance. Although only HR fl-E6 has the ability to transform its target cell, HPV18 E6^*^ protein binds to fl-E6 preventing p53 degradation (43, 44). Furthermore, contrarily to what is seen with HPV16 E6, binding of E6^*^ to procaspase-8 results in its stabilization and may sensitize cells to apoptosis (45). Also, E6^*^II sensitizes C33A cell to cisplatin-induced apoptosis (46). Moreover, HPV16 E6^*^ reduces tumor formation in cervical carcinoma xenografts in mice, pointing to anti-oncogenic characteristics of E6^*^ (47). In contrast, Rosenberger et al. propose that during the first stages of viral infection of the basal keratinocytes, HPV might require high levels of fl-E6 to inhibit apoptosis through p53 degradation, and that afterwards with increasing cell differentiation, HPV would express E7, and therefore E6^*^, to overcome reduced cell proliferation (35). Additionally, E6^*^ may participate in virus-induced mutagenesis by increasing oxidative stress and DNA damage (48). All these studies must be put together and corroborated *in vivo* to understand the mechanisms that regulate the splicing of HPV mRNAs and the biological significance of the switch from one transcript to another.

Although many studies have focused on IL-6 and its involvement in cervical carcinogenesis, there are few reports on IL-6R and its implication in CC. In the present work, we demonstrate that IL-6 receptor expression is increased in CC cell lines and in cervical tissues derived from women with squamous cell carcinoma. Interestingly, IL-6R expression was also positive in the proliferative basal layer of the cervical epithelium in control tissues, suggesting that IL-6R might be expressed in proliferative and tumoral keratinocytes, and that the high IL-6 expression in CC could be acting in an autocrine and paracrine manner in keratinocytes and not only in immune cells. In line with our results, it has been recently demonstrated that IL-6R is overexpressed CC tissue compared to normal control tissue (49). HR HPV infected keratinocytes are therefore capable of secreting high levels of IL-6 due in part to the presence of viral oncoproteins, and also expressing IL-6R and be receptive to IL-6 signaling.

Finally, it is worth mentioning that IL-6 synthesis is strictly regulated both transcriptionally and post-transcriptionally (15). Transcription factors like NF-IL6 and NF-κB activate IL-6 transcription (50). In contrast, Rb and p53 proteins repress IL-6 promoter, and mutant p53 has the opposite effect contributing to IL-6 overexpression (25, 26). HaCaT immortalized keratinocytes have dysfunctional mutated p53 that in our study model is insensitive to E6^*^-targeted degradation (22, 23). In this regard, IL-6 up-modulation by E6^*^ observed in HaCaT cells is mediated independently of p53 degradation. This conclusion is also supported by the upregulation of IL-6 after silencing wt p53 in immortalized keratinocytes that is not as strong as in the presence of E6E7, in both mutated p53 and wt p53 keratinocytes (20). Therefore, this process could be modulated by E6 trough induction of p53 degradation but also by a mechanism independent of p53.

In conclusion, HPV oncoproteins are multifunctional and in this work we show that IL-6 is one of their multiple targets. Indeed, E5, E6, and E7 are involved by many mechanisms in the development of chronic inflammation associated with cervical carcinogenesis (51). Both HR and LR E6 and E7 proteins upregulate IL-6 expression in keratinocytes, especially HR E6^*^ isoforms, and IL-6 is also overexpressed in CIN1 samples compared to control cervical samples without lesion. IL-6R is also overexpressed in tumoral proliferative keratinocytes in CC tissues which are then receptive to proliferative signals through IL-6 signaling. Since many studies have reported E6^*^ as the predominant transcript in malignant lesions and CC, this E6 truncated form may participate in the maintenance of the chronic inflammatory microenvironment through IL-6 overexpression. Further studies must unravel if upregulation of IL-6 in keratinocytes is correlated with keratinocyte transformation during the first stages of viral infection or with proliferation and malignancy that promote carcinogenesis.

## Data Availability

All datasets generated for this study are included in the manuscript and/or the Supplementary Files.

## Ethics Statement

Cervical samples were collected from women who attended a medical examination at the Clinic of Dysplasia in the Gynecology and Obstetrics Hospital- Centro Médico Nacional de Occidente (CMNO-IMSS) in Guadalajara, Jalisco, Mexico. Before sampling, all participants were informed about this research protocol and decided to participate voluntarily; all of them signed informed consent. All procedures performed were in accordance with the ethical standards and were approved by the National Scientific Research Committee of IMSS (CNIC) with the Register number: R-2012-785-090.

## Author Contributions

CA-I cloned all HPV16, 18, 62, and 84 proteins, developed the HaCaT transduced cell lines, conducted the transient and stable transfections, performed the IL-6 and p53 expression analysis, and wrote the first draft of the manuscript. LL-T, PO-L, and WR-C were involved in patient interviews and cervical sample recruitment. AM-P performed the Linear Array HPV test and qPCR assays with the cervical samples. AP-S was involved in the immunohistochemistry analysis. AA-L and LJ-S designed the study, supervised all experiments and analyses, and completed the manuscript. All authors read and approved the final manuscript.

### Conflict of Interest Statement

The authors declare that the research was conducted in the absence of any commercial or financial relationships that could be construed as a potential conflict of interest.

## Supplementary Material

Table S1 Primers and experimental conditions used for PCR amplifications.

Figure S1 p53 and IL-6 expression in E6 transduced HaCaT cells compared to HaCaT-pLVX control cells, after p53 wt transfection. **(A)** p53 mRNA relative expression in HaCaT-pLVX, −16E6^*^, −18E6^*^, −62E6, and −84E6 cells transfected with pcDNA3-p53wt compared to cells transfected with empty pcDNA3 vector. **(B)** IL-6 mRNA expression in the same cells transfected with pcDNA3-p53wt compared to cells transfected with pcDNA3 empty vector. Actin was used as reference gene and error bars represent standard deviation. Results from three independent experiments are shown in this Figure. pcDNA3 and pcDNA3-p53wt were transfected in the cells using X-tremeGENE HP DNA Transfection Reagent (06365752001, Roche) and RNA was extracted 24 h post-transfection.

## Abbreviations

CC: cervical cancer
CIN1: Cervical Intraepithelial Neoplasia grade 1
HR-HPV: High-Risk Human Papillomavirus
LR-HPV: Low-Risk Human Papillomavirus
fl-E6: full length E6
IL-6R: IL-6 receptor.
