# Supplementary material for: E6/E7 and E6* From HPV16 and HPV18 Upregulate IL-6 Expression Independently of p53 in Keratinocytes
Source: Front Immunol. 2019 Jul 23;10:1676. doi: 10.3389/fimmu.2019.01676 (PMC6664019; doi:10.3389/fimmu.2019.01676)
Supplement: Table S1 — Primers and experimental conditions used for PCR amplifications. [file Table_1.DOCX]

| Table S1. Primers and experimental conditions used for PCR amplifications. | | | | |
| --- | --- | --- | --- | --- |
| Primer | Sequence | Product Length | Tm  (ºC) | Elongation time (s) |
| HPV16 E5 | F: 5' tggatttatgtctatatgacaa 3'  R: 5' attatgtaattaaaaagcgtg 3' | 268 bp | 55 | 12 |
| HPV16 E6/E6* | F: 5' cagacattttatgcaccaaa 3'  R: 5' ctccatgcatgattacagc 3' | 499 bp/317 bp/200 bp | 58 | 22 |
| HPV16 E7 | F: 5' tagagaaacccagctgtaatca 3'  R: 5' aggatcagccatggtagattat 3' | 336 bp | 60 | 16 |
| HPV18 E5 | F: 5' tgctgtagtaccaatatgtta 3'  R: 5' aaaacaacctatacaattactg 3' | 253 bp | 55 | 12 |
| HPV18 E6/E6* | F: 5' aatactatggcgcgctttga 3'  R: 5' ttgccttaggtccatgcatact 3' | 510 bp/328 bp | 65 | 22 |
| HPV18 E7 | F: 5' cgcagagaaacacaagtataat 3'  R: 5' gatcagccattgttgctta 3' | 363 bp | 60 | 16 |
| HPV62 E5 | F: 5' tgcaggtgtttagtaggtatg 3'  R: 5' gtacaacgctggtagttacaat 3' | 174 bp | 58 | 8 |
| HPV62 E6 | F: 5' ggtcagcacagtagcaatgact 3'  R: 5' cgggacgctcttgtaggac 3' | 490 bp | 63 | 21 |
| HPV62 E7 | F: 5' caggagtgtggacaggacggta 3'  R: 5' gcatcggccatgtcacttatg 3' | 347 bp | 67 | 15 |
| HPV84 E5 | F: 5' agtcccggtcctacctatta 3'  R: 5' gcactttaaaactgcataacac 3' | 168 bp | 58 | 8 |
| HPV84 E6 | F: 5' atataaagggcagcaaacggta 3'  R: 5' atatcctttaacgttggcgtct 3' | 487 bp | 63 | 21 |
| HPV84 E7 | F: 5' gctgcaactgtagggtaaca 3'  R: 5' ttcaggtgactctgccatatta 3' | 329 bp | 61 | 14 |
| ACTB | F: 5' tccgcaaagacctgtacg 3'  R: 5' aagaaagggtgtaacgcaacta 3' | 298 bp | 60 | 12 |
| GAPDH | F: 5' cactgccacccagaagactgtg 3'  R: 5' tgtaggccatgaggtccaccac 3' | 449 bp | 60 | 23 |
| RPL32 | F: 5' gcattgacaacagggttcgtag 3'  R: 5' atttaaacagaaaacgtgcaca 3' | 320 bp | 60 | 12 |
| RPLP0 | F: 5' cctcatatccgggggaatgtg 3'  R: 5' gcagcagctggcaccttattg 3' | 95 bp | 58 | 4 |
| IL-6 | F: 5' tacaaaagtcctgatccagttc 3'  R: 5' aagaaggaatgcccattaac 3' | 245 bp | 60 | 10 |
| IL6RA | F: 5' ggcacgccttggacagaatcc 3'  R: 5' ccgcagcttccacgtcttctt 3' | 252 bp | 60 | 12 |
